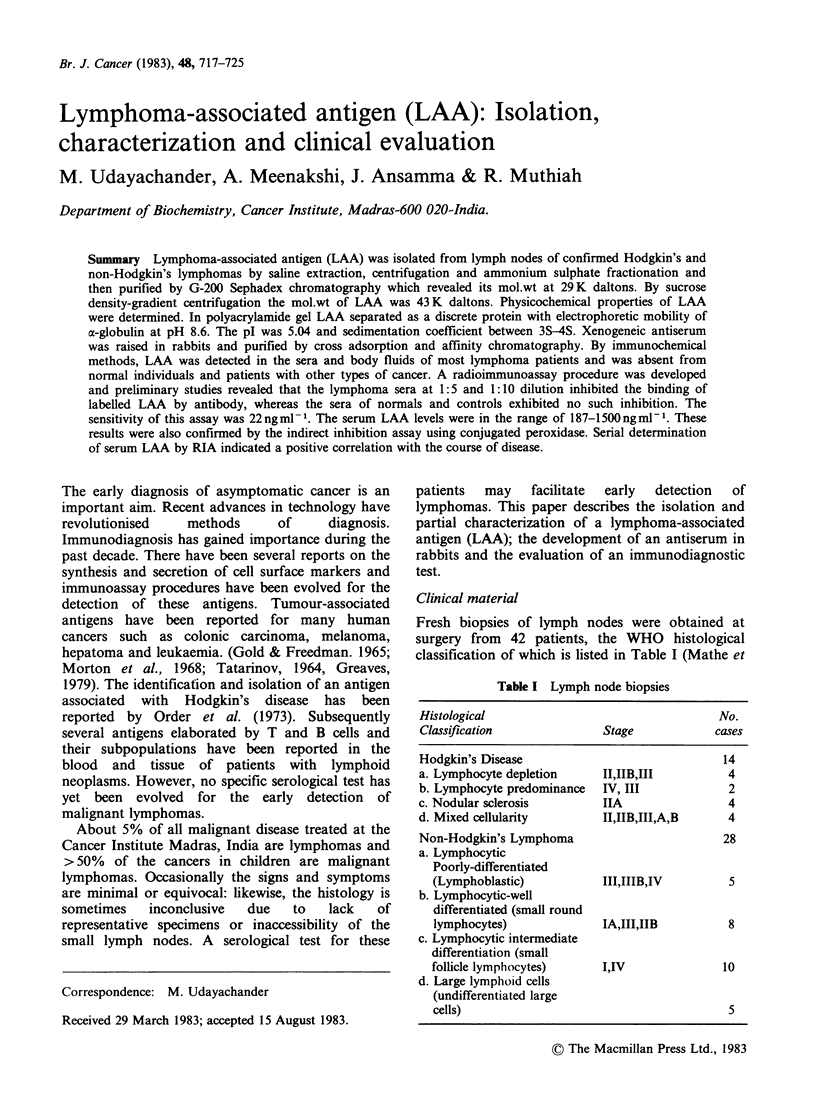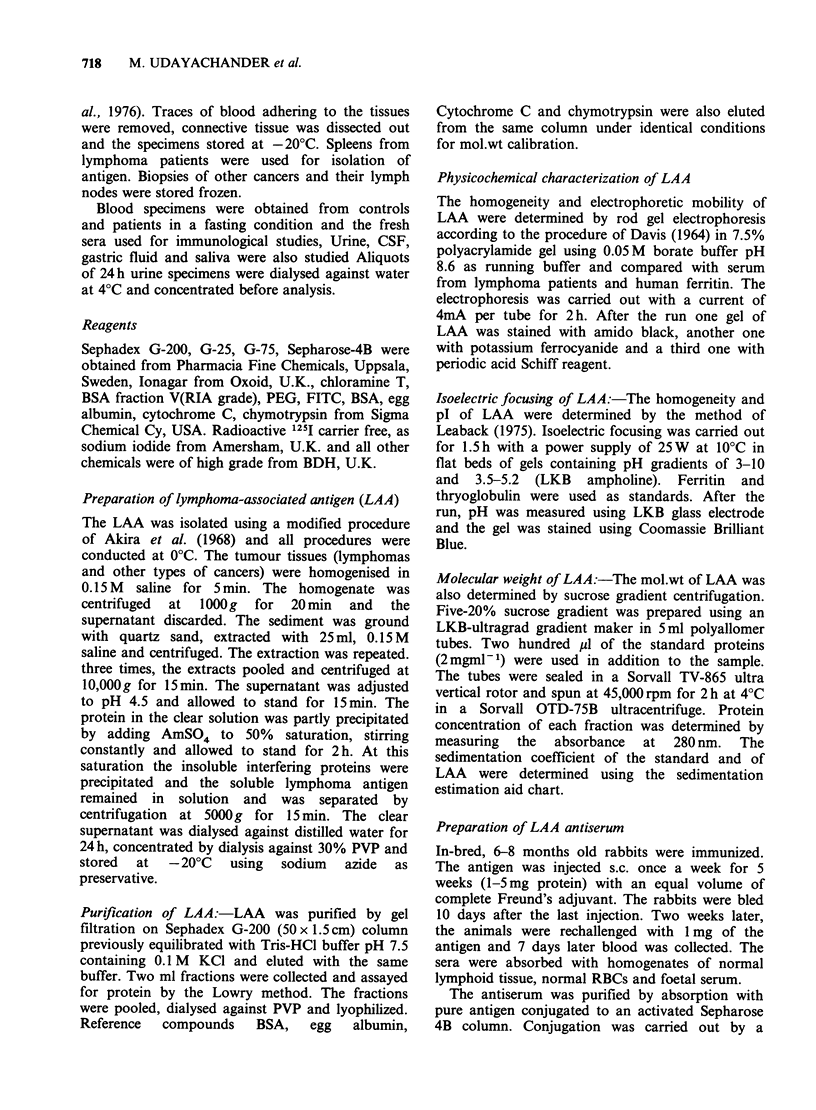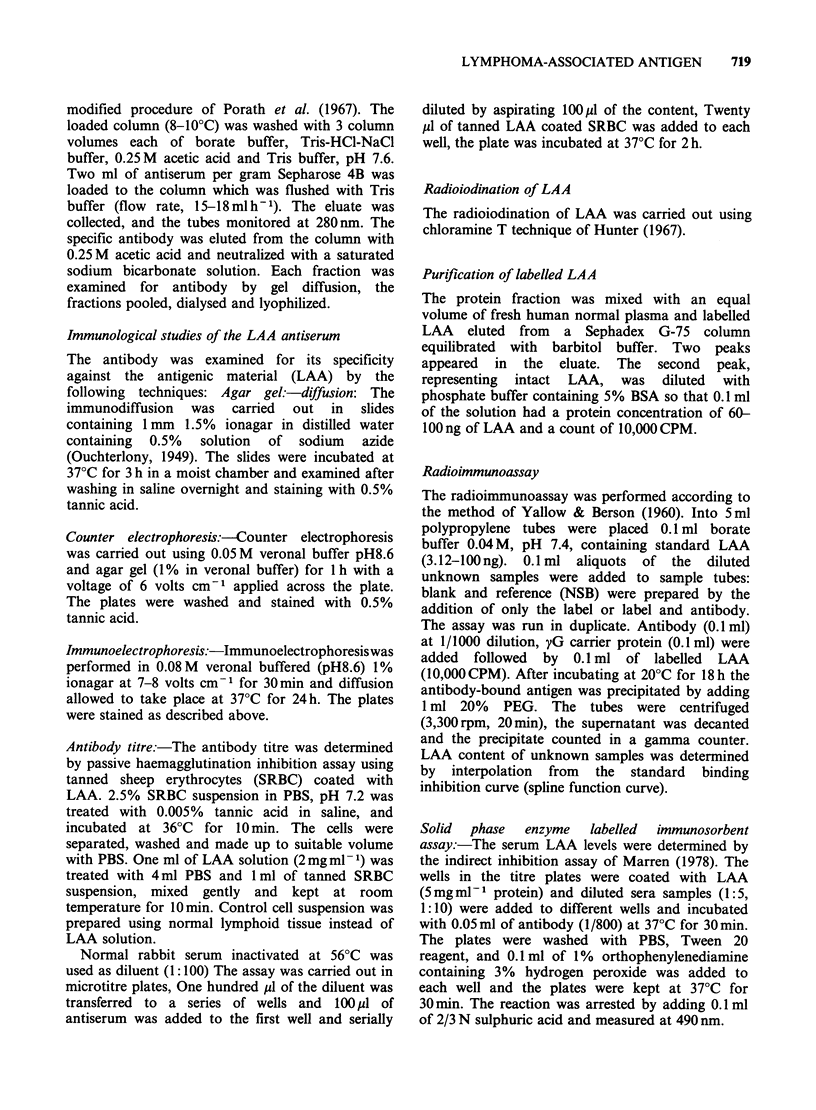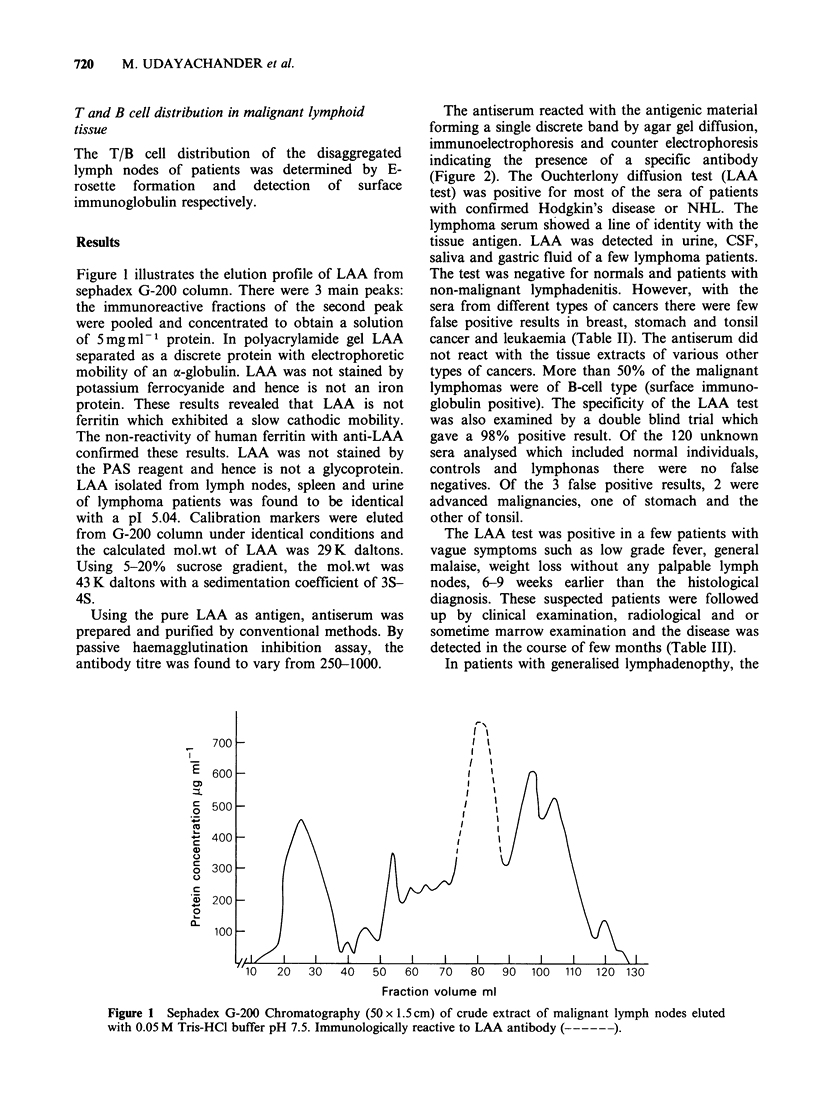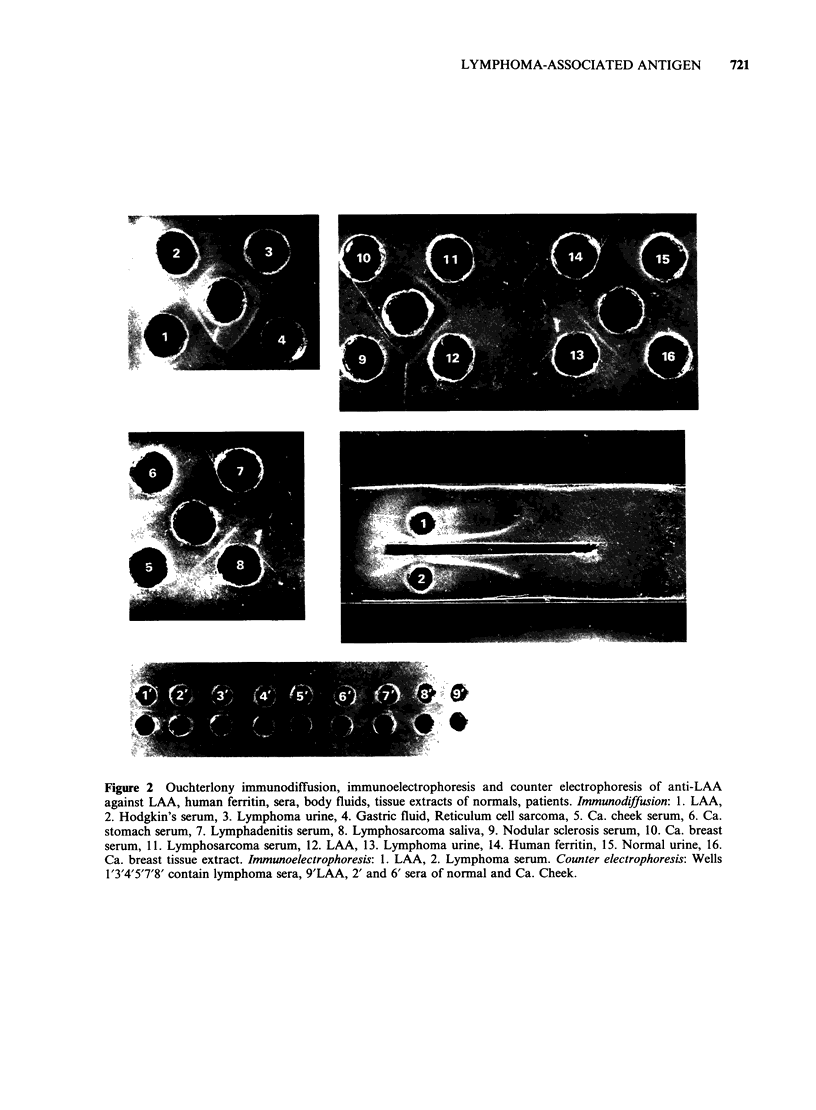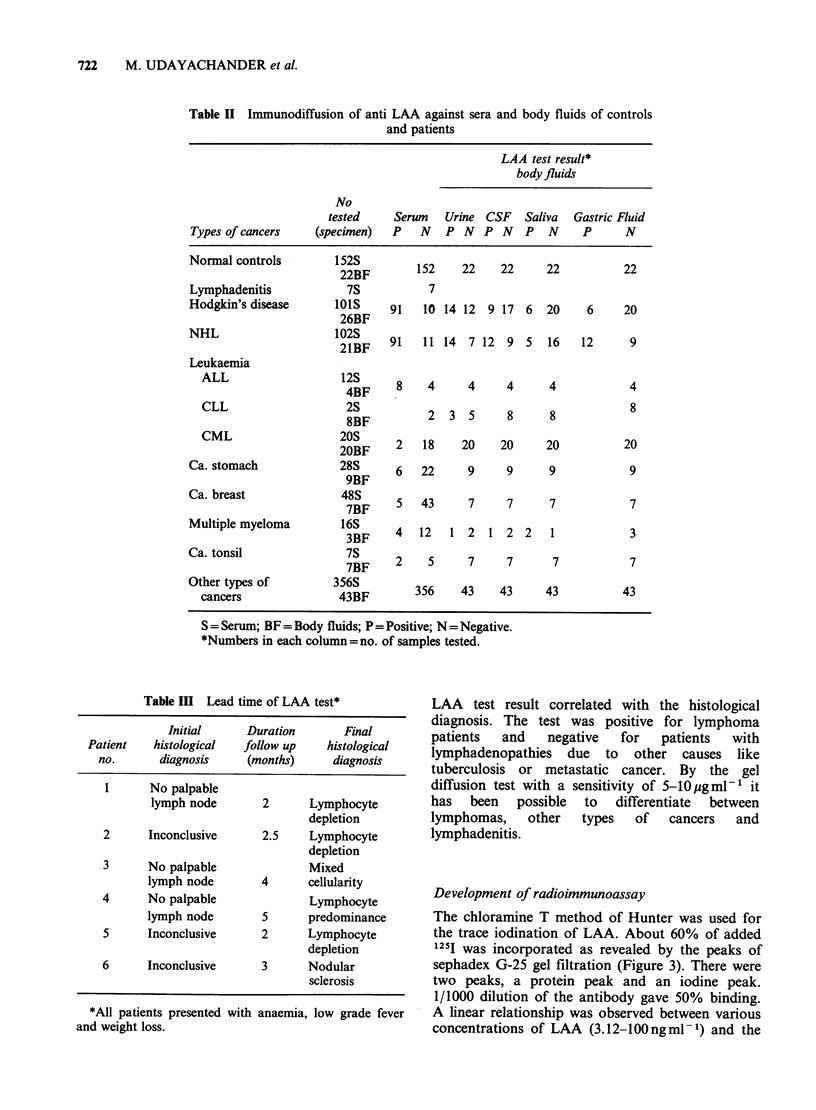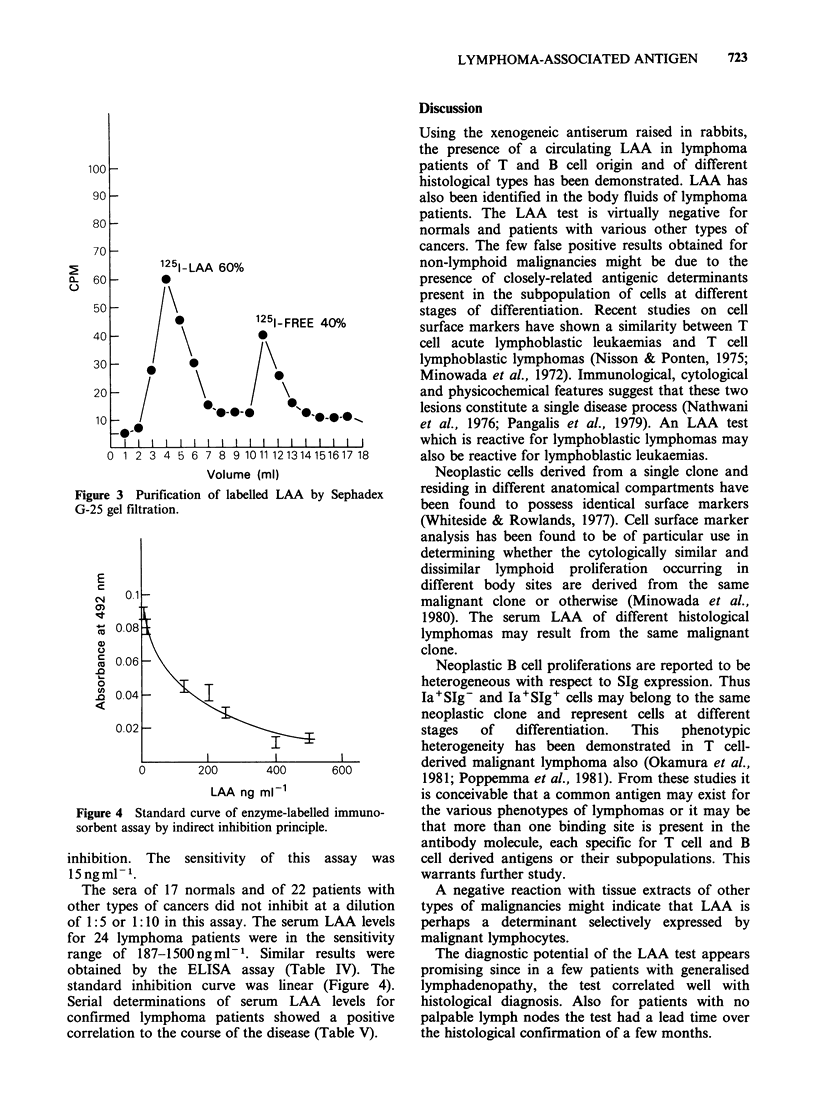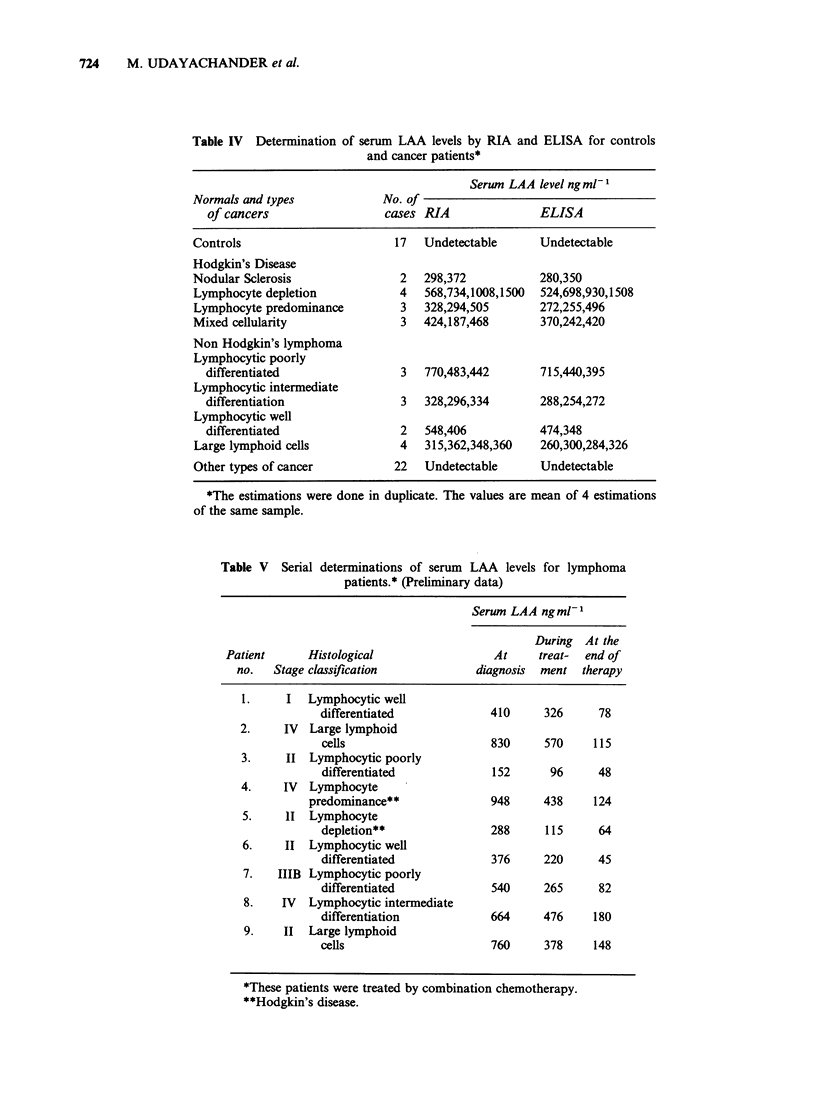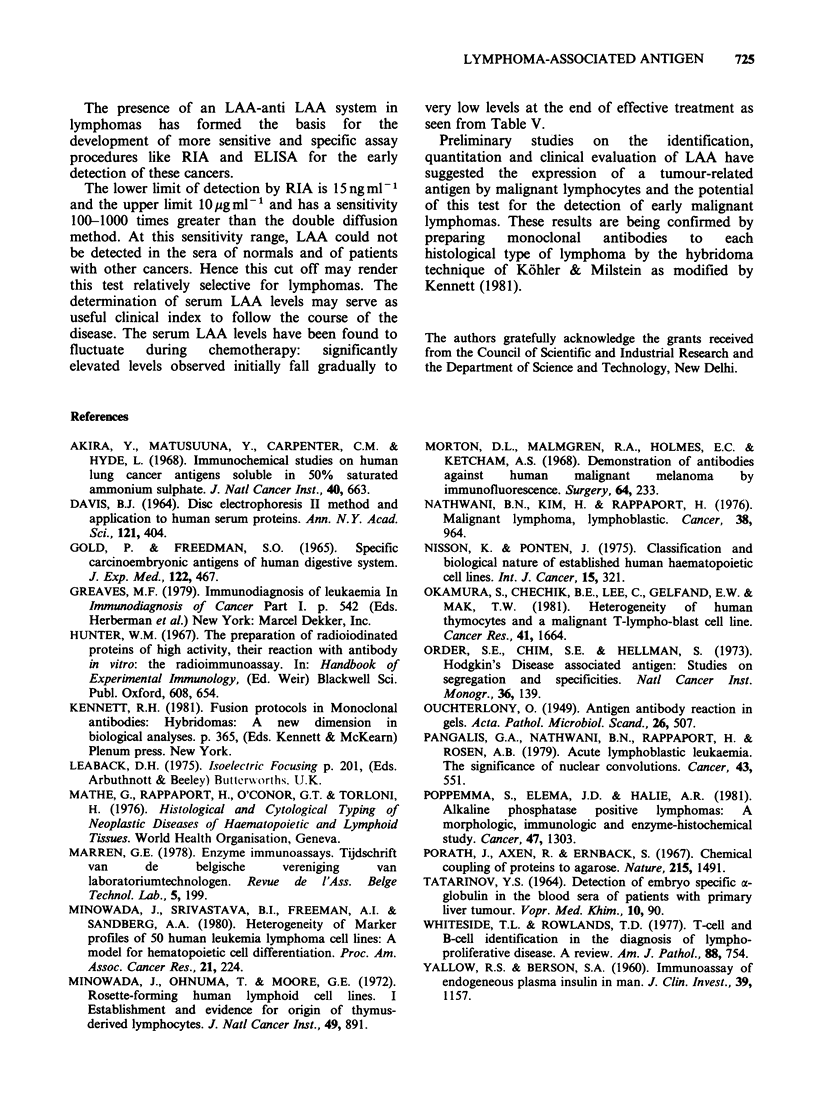# Lymphoma-associated antigen (LAA): isolation, characterization and clinical evaluation.

**DOI:** 10.1038/bjc.1983.255

**Published:** 1983-11

**Authors:** M. Udayachander, A. Meenakshi, J. Ansamma, R. Muthiah

## Abstract

**Images:**


*Br. J. Cancer* (1983), **48**, 717–725

### Lymphoma-associated antigen (LAA): Isolation, characterization and clinical evaluation

M. Udayachander, A. Meenakshi, J. Ansamma & R. Muthiah

*Department of Biochemistry, Cancer Institute, Madras-600 020-India.*

**Summary** Lymphoma-associated antigen (LAA) was isolated from lymph nodes of confirmed Hodgkin's and non-Hodgkin's lymphomas by saline extraction, centrifugation and ammonium sulphate fractionation and then purified by G-200 Sephadex chromatography which revealed its mol.wt at 29 K daltons. By sucrose density-gradient centrifugation the mol.wt of LAA was 43 K daltons. Physicochemical properties of LAA were determined. In polyacrylamide gel LAA separated as a discrete protein with electrophoretic mobility of $\alpha$-globulin at pH 8.6. The pI was 5.04 and sedimentation coefficient between 3S–4S. Xenogeneic antiserum was raised in rabbits and purified by cross adsorption and affinity chromatography. By immunochemical methods, LAA was detected in the sera and body fluids of most lymphoma patients and was absent from normal individuals and patients with other types of cancer. A radioimmunoassay procedure was developed and preliminary studies revealed that the lymphoma sera at 1:5 and 1:10 dilution inhibited the binding of labelled LAA by antibody, whereas the sera of normals and controls exhibited no such inhibition. The sensitivity of this assay was 22 ng ml$^{-1}$. The serum LAA levels were in the range of 187–1500 ng ml$^{-1}$. These results were also confirmed by the indirect inhibition assay using conjugated peroxidase. Serial determination of serum LAA by RIA indicated a positive correlation with the course of disease.

The early diagnosis of asymptomatic cancer is an important aim. Recent advances in technology have revolutionised methods of diagnosis. Immunodiagnosis has gained importance during the past decade. There have been several reports on the synthesis and secretion of cell surface markers and immunoassay procedures have been evolved for the detection of these antigens. Tumour-associated antigens have been reported for many human cancers such as colonic carcinoma, melanoma, hepatoma and leukaemia. (Gold & Freedman. 1965; Morton *et al.,* 1968; Tatarinov, 1964, Greaves, 1979). The identification and isolation of an antigen associated with Hodgkin's disease has been reported by Order *et al.* (1973). Subsequently several antigens elaborated by T and B cells and their subpopulations have been reported in the blood and tissue of patients with lymphoid neoplasms. However, no specific serological test has yet been evolved for the early detection of malignant lymphomas.

About 5% of all malignant disease treated at the Cancer Institute Madras, India are lymphomas and >50% of the cancers in children are malignant lymphomas. Occasionally the signs and symptoms are minimal or equivocal: likewise, the histology is sometimes inconclusive due to lack of representative specimens or inaccessibility of the small lymph nodes. A serological test for these patients may facilitate early detection of lymphomas. This paper describes the isolation and partial characterization of a lymphoma-associated antigen (LAA); the development of an antiserum in rabbits and the evaluation of an immunodiagnostic test.

### *Clinical material*

Fresh biopsies of lymph nodes were obtained at surgery from 42 patients, the WHO histological classification of which is listed in Table I (Mathe *et*

**Table I** Lymph node biopsies

| *Histological Classification* | *Stage* | *No. cases* |
|---|---|---|
| Hodgkin's Disease | | 14 |
| a. Lymphocyte depletion | II,IIB,III | 4 |
| b. Lymphocyte predominance | IV, III | 2 |
| c. Nodular sclerosis | IIA | 4 |
| d. Mixed cellularity | II,IIB,III,A,B | 4 |
| Non-Hodgkin's Lymphoma | | 28 |
| a. Lymphocytic Poorly-differentiated (Lymphoblastic) | III,IIIB,IV | 5 |
| b. Lymphocytic-well differentiated (small round lymphocytes) | IA,III,IIB | 8 |
| c. Lymphocytic intermediate differentiation (small follicle lymphocytes) | I,IV | 10 |
| d. Large lymphoid cells (undifferentiated large cells) | | 5 |

Correspondence: M. Udayachander

Received 29 March 1983; accepted 15 August 1983.

© The Macmillan Press Ltd., 1983

al., 1976). Traces of blood adhering to the tissues were removed, connective tissue was dissected out and the specimens stored at −20°C. Spleens from lymphoma patients were used for isolation of antigen. Biopsies of other cancers and their lymph nodes were stored frozen.

Blood specimens were obtained from controls and patients in a fasting condition and the fresh sera used for immunological studies, Urine, CSF, gastric fluid and saliva were also studied Aliquots of 24 h urine specimens were dialysed against water at 4°C and concentrated before analysis.

*Reagents*

Sephadex G-200, G-25, G-75, Sepharose-4B were obtained from Pharmacia Fine Chemicals, Uppsala, Sweden, Ionagar from Oxoid, U.K., chloramine T, BSA fraction V(RIA grade), PEG, FITC, BSA, egg albumin, cytochrome C, chymotrypsin from Sigma Chemical Cy, USA. Radioactive $^{125}$I carrier free, as sodium iodide from Amersham, U.K. and all other chemicals were of high grade from BDH, U.K.

*Preparation of lymphoma-associated antigen (LAA)*

The LAA was isolated using a modified procedure of Akira *et al.* (1968) and all procedures were conducted at 0°C. The tumour tissues (lymphomas and other types of cancers) were homogenised in 0.15 M saline for 5 min. The homogenate was centrifuged at 1000 *g* for 20 min and the supernatant discarded. The sediment was ground with quartz sand, extracted with 25 ml, 0.15 M saline and centrifuged. The extraction was repeated. three times, the extracts pooled and centrifuged at 10,000 *g* for 15 min. The supernatant was adjusted to pH 4.5 and allowed to stand for 15 min. The protein in the clear solution was partly precipitated by adding $AmSO_4$ to 50% saturation, stirring constantly and allowed to stand for 2 h. At this saturation the insoluble interfering proteins were precipitated and the soluble lymphoma antigen remained in solution and was separated by centrifugation at 5000 *g* for 15 min. The clear supernatant was dialysed against distilled water for 24 h, concentrated by dialysis against 30% PVP and stored at −20°C using sodium azide as preservative.

*Purification of LAA:*—LAA was purified by gel filtration on Sephadex G-200 (50 × 1.5 cm) column previously equilibrated with Tris-HCl buffer pH 7.5 containing 0.1 M KCl and eluted with the same buffer. Two ml fractions were collected and assayed for protein by the Lowry method. The fractions were pooled, dialysed against PVP and lyophilized. Reference compounds BSA, egg albumin, Cytochrome C and chymotrypsin were also eluted from the same column under identical conditions for mol.wt calibration.

*Physicochemical characterization of LAA*

The homogeneity and electrophoretic mobility of LAA were determined by rod gel electrophoresis according to the procedure of Davis (1964) in 7.5% polyacrylamide gel using 0.05 M borate buffer pH 8.6 as running buffer and compared with serum from lymphoma patients and human ferritin. The electrophoresis was carried out with a current of 4mA per tube for 2 h. After the run one gel of LAA was stained with amido black, another one with potassium ferrocyanide and a third one with periodic acid Schiff reagent.

*Isoelectric focusing of LAA:*—The homogeneity and pI of LAA were determined by the method of Leaback (1975). Isoelectric focusing was carried out for 1.5 h with a power supply of 25 W at 10°C in flat beds of gels containing pH gradients of 3–10 and 3.5–5.2 (LKB ampholine). Ferritin and thryoglobulin were used as standards. After the run, pH was measured using LKB glass electrode and the gel was stained using Coomassie Brilliant Blue.

*Molecular weight of LAA:*—The mol.wt of LAA was also determined by sucrose gradient centrifugation. Five-20% sucrose gradient was prepared using an LKB-ultragrad gradient maker in 5 ml polyallomer tubes. Two hundred $\mu$l of the standard proteins (2 mgml$^{-1}$) were used in addition to the sample. The tubes were sealed in a Sorvall TV-865 ultra vertical rotor and spun at 45,000 rpm for 2 h at 4°C in a Sorvall OTD-75B ultracentrifuge. Protein concentration of each fraction was determined by measuring the absorbance at 280 nm. The sedimentation coefficient of the standard and of LAA were determined using the sedimentation estimation aid chart.

*Preparation of LAA antiserum*

In-bred, 6–8 months old rabbits were immunized. The antigen was injected s.c. once a week for 5 weeks (1–5 mg protein) with an equal volume of complete Freund's adjuvant. The rabbits were bled 10 days after the last injection. Two weeks later, the animals were rechallenged with 1 mg of the antigen and 7 days later blood was collected. The sera were absorbed with homogenates of normal lymphoid tissue, normal RBCs and foetal serum.

The antiserum was purified by absorption with pure antigen conjugated to an activated Sepharose 4B column. Conjugation was carried out by a modified procedure of Porath *et al.* (1967). The loaded column (8–10°C) was washed with 3 column volumes each of borate buffer, Tris-HCl-NaCl buffer, 0.25 M acetic acid and Tris buffer, pH 7.6. Two ml of antiserum per gram Sepharose 4B was loaded to the column which was flushed with Tris buffer (flow rate, 15–18 ml $h^{-1}$). The eluate was collected, and the tubes monitored at 280 nm. The specific antibody was eluted from the column with 0.25 M acetic acid and neutralized with a saturated sodium bicarbonate solution. Each fraction was examined for antibody by gel diffusion, the fractions pooled, dialysed and lyophilized.

*Immunological studies of the LAA antiserum*

The antibody was examined for its specificity against the antigenic material (LAA) by the following techniques: *Agar gel:—diffusion*: The immunodiffusion was carried out in slides containing 1 mm 1.5% ionagar in distilled water containing 0.5% solution of sodium azide (Ouchterlony, 1949). The slides were incubated at 37°C for 3 h in a moist chamber and examined after washing in saline overnight and staining with 0.5% tannic acid.

*Counter electrophoresis:*—Counter electrophoresis was carried out using 0.05 M veronal buffer pH8.6 and agar gel (1% in veronal buffer) for 1 h with a voltage of 6 volts $cm^{-1}$ applied across the plate. The plates were washed and stained with 0.5% tannic acid.

*Immunoelectrophoresis:*—Immunoelectrophoresis was performed in 0.08 M veronal buffered (pH8.6) 1% ionagar at 7–8 volts $cm^{-1}$ for 30 min and diffusion allowed to take place at 37°C for 24 h. The plates were stained as described above.

*Antibody titre:*—The antibody titre was determined by passive haemagglutination inhibition assay using tanned sheep erythrocytes (SRBC) coated with LAA. 2.5% SRBC suspension in PBS, pH 7.2 was treated with 0.005% tannic acid in saline, and incubated at 36°C for 10 min. The cells were separated, washed and made up to suitable volume with PBS. One ml of LAA solution (2 mg $ml^{-1}$) was treated with 4 ml PBS and 1 ml of tanned SRBC suspension, mixed gently and kept at room temperature for 10 min. Control cell suspension was prepared using normal lymphoid tissue instead of LAA solution.

Normal rabbit serum inactivated at 56°C was used as diluent (1:100) The assay was carried out in microtitre plates, One hundred $\mu l$ of the diluent was transferred to a series of wells and 100 $\mu l$ of antiserum was added to the first well and serially diluted by aspirating 100 $\mu l$ of the content, Twenty $\mu l$ of tanned LAA coated SRBC was added to each well, the plate was incubated at 37°C for 2 h.

*Radioiodination of LAA*

The radioiodination of LAA was carried out using chloramine T technique of Hunter (1967).

*Purification of labelled LAA*

The protein fraction was mixed with an equal volume of fresh human normal plasma and labelled LAA eluted from a Sephadex G-75 column equilibrated with barbitol buffer. Two peaks appeared in the eluate. The second peak, representing intact LAA, was diluted with phosphate buffer containing 5% BSA so that 0.1 ml of the solution had a protein concentration of 60–100 ng of LAA and a count of 10,000 CPM.

*Radioimmunoassay*

The radioimmunoassay was performed according to the method of Yallow & Berson (1960). Into 5 ml polypropylene tubes were placed 0.1 ml borate buffer 0.04 M, pH 7.4, containing standard LAA (3.12–100 ng). 0.1 ml aliquots of the diluted unknown samples were added to sample tubes: blank and reference (NSB) were prepared by the addition of only the label or label and antibody. The assay was run in duplicate. Antibody (0.1 ml) at 1/1000 dilution, $\gamma$G carrier protein (0.1 ml) were added followed by 0.1 ml of labelled LAA (10,000 CPM). After incubating at 20°C for 18 h the antibody-bound antigen was precipitated by adding 1 ml 20% PEG. The tubes were centrifuged (3,300 rpm, 20 min), the supernatant was decanted and the precipitate counted in a gamma counter. LAA content of unknown samples was determined by interpolation from the standard binding inhibition curve (spline function curve).

*Solid phase enzyme labelled immunosorbent assay:*—The serum LAA levels were determined by the indirect inhibition assay of Marren (1978). The wells in the titre plates were coated with LAA (5 mg $ml^{-1}$ protein) and diluted sera samples (1:5, 1:10) were added to different wells and incubated with 0.05 ml of antibody (1/800) at 37°C for 30 min. The plates were washed with PBS, Tween 20 reagent, and 0.1 ml of 1% orthophenylenediamine containing 3% hydrogen peroxide was added to each well and the plates were kept at 37°C for 30 min. The reaction was arrested by adding 0.1 ml of 2/3 N sulphuric acid and measured at 490 nm.

*T and B cell distribution in malignant lymphoid tissue*

The T/B cell distribution of the disaggregated lymph nodes of patients was determined by E-rosette formation and detection of surface immunoglobulin respectively.

### Results

Figure 1 illustrates the elution profile of LAA from sephadex G-200 column. There were 3 main peaks: the immunoreactive fractions of the second peak were pooled and concentrated to obtain a solution of $5\,mg\,ml^{-1}$ protein. In polyacrylamide gel LAA separated as a discrete protein with electrophoretic mobility of an $\alpha$-globulin. LAA was not stained by potassium ferrocyanide and hence is not an iron protein. These results revealed that LAA is not ferritin which exhibited a slow cathodic mobility. The non-reactivity of human ferritin with anti-LAA confirmed these results. LAA was not stained by the PAS reagent and hence is not a glycoprotein. LAA isolated from lymph nodes, spleen and urine of lymphoma patients was found to be identical with a pI 5.04. Calibration markers were eluted from G-200 column under identical conditions and the calculated mol.wt of LAA was 29 K daltons. Using 5–20% sucrose gradient, the mol.wt was 43 K daltons with a sedimentation coefficient of 3S–4S.

Using the pure LAA as antigen, antiserum was prepared and purified by conventional methods. By passive haemagglutination inhibition assay, the antibody titre was found to vary from 250–1000.

The antiserum reacted with the antigenic material forming a single discrete band by agar gel diffusion, immunoelectrophoresis and counter electrophoresis indicating the presence of a specific antibody (Figure 2). The Ouchterlony diffusion test (LAA test) was positive for most of the sera of patients with confirmed Hodgkin's disease or NHL. The lymphoma serum showed a line of identity with the tissue antigen. LAA was detected in urine, CSF, saliva and gastric fluid of a few lymphoma patients. The test was negative for normals and patients with non-malignant lymphadenitis. However, with the sera from different types of cancers there were few false positive results in breast, stomach and tonsil cancer and leukaemia (Table II). The antiserum did not react with the tissue extracts of various other types of cancers. More than 50% of the malignant lymphomas were of B-cell type (surface immunoglobulin positive). The specificity of the LAA test was also examined by a double blind trial which gave a 98% positive result. Of the 120 unknown sera analysed which included normal individuals, controls and lymphonas there were no false negatives. Of the 3 false positive results, 2 were advanced malignancies, one of stomach and the other of tonsil.

The LAA test was positive in a few patients with vague symptoms such as low grade fever, general malaise, weight loss without any palpable lymph nodes, 6–9 weeks earlier than the histological diagnosis. These suspected patients were followed up by clinical examination, radiological and or sometime marrow examination and the disease was detected in the course of few months (Table III).

In patients with generalised lymphadenopthy, the

**Figure 1** Sephadex G-200 Chromatography (50 × 1.5 cm) of crude extract of malignant lymph nodes eluted with 0.05 M Tris-HCl buffer pH 7.5. Immunologically reactive to LAA antibody (------).

**Figure 2** Ouchterlony immunodiffusion, immunoelectrophoresis and counter electrophoresis of anti-LAA against LAA, human ferritin, sera, body fluids, tissue extracts of normals, patients. *Immunodiffusion*: 1. LAA, 2. Hodgkin's serum, 3. Lymphoma urine, 4. Gastric fluid, Reticulum cell sarcoma, 5. Ca. cheek serum, 6. Ca. stomach serum, 7. Lymphadenitis serum, 8. Lymphosarcoma saliva, 9. Nodular sclerosis serum, 10. Ca. breast serum, 11. Lymphosarcoma serum, 12. LAA, 13. Lymphoma urine, 14. Human ferritin, 15. Normal urine, 16. Ca. breast tissue extract. *Immunoelectrophoresis*: 1. LAA, 2. Lymphoma serum. *Counter electrophoresis*: Wells 1′3′4′5′7′8′ contain lymphoma sera, 9′LAA, 2′ and 6′ sera of normal and Ca. Cheek.

**Table II** Immunodiffusion of anti LAA against sera and body fluids of controls and patients

| | | *LAA test result** *body fluids* | | | | | | | | | |
|---|---|---|---|---|---|---|---|---|---|---|---|
| *Types of cancers* | *No tested (specimen)* | *Serum* *P* | *Serum* *N* | *Urine* *P* | *Urine* *N* | *CSF* *P* | *CSF* *N* | *Saliva* *P* | *Saliva* *N* | *Gastric Fluid* *P* | *Gastric Fluid* *N* |
| Normal controls | 152S<br>22BF | | 152 | | 22 | | 22 | | 22 | | 22 |
| Lymphadenitis | 7S | | 7 | | | | | | | | |
| Hodgkin's disease | 101S<br>26BF | 91 | 10 | 14 | 12 | 9 | 17 | 6 | 20 | 6 | 20 |
| NHL | 102S<br>21BF | 91 | 11 | 14 | 7 | 12 | 9 | 5 | 16 | 12 | 9 |
| Leukaemia | | | | | | | | | | | |
| ALL | 12S<br>4BF | 8 | 4 | | 4 | | 4 | | 4 | | 4 |
| CLL | 2S<br>8BF | | 2 | 3 | 5 | | 8 | | 8 | | 8 |
| CML | 20S<br>20BF | 2 | 18 | | 20 | | 20 | | 20 | | 20 |
| Ca. stomach | 28S<br>9BF | 6 | 22 | | 9 | | 9 | | 9 | | 9 |
| Ca. breast | 48S<br>7BF | 5 | 43 | | 7 | | 7 | | 7 | | 7 |
| Multiple myeloma | 16S<br>3BF | 4 | 12 | 1 | 2 | 1 | 2 | 2 | 1 | | 3 |
| Ca. tonsil | 7S<br>7BF | 2 | 5 | | 7 | | 7 | | 7 | | 7 |
| Other types of cancers | 356S<br>43BF | | 356 | | 43 | | 43 | | 43 | | 43 |

S = Serum; BF = Body fluids; P = Positive; N = Negative.
*Numbers in each column = no. of samples tested.

**Table III** Lead time of LAA test*

| *Patient no.* | *Initial histological diagnosis* | *Duration follow up (months)* | *Final histological diagnosis* |
|---|---|---|---|
| 1 | No palpable lymph node | 2 | Lymphocyte depletion |
| 2 | Inconclusive | 2.5 | Lymphocyte depletion |
| 3 | No palpable lymph node | 4 | Mixed cellularity |
| 4 | No palpable lymph node | 5 | Lymphocyte predominance |
| 5 | Inconclusive | 2 | Lymphocyte depletion |
| 6 | Inconclusive | 3 | Nodular sclerosis |

*All patients presented with anaemia, low grade fever and weight loss.

LAA test result correlated with the histological diagnosis. The test was positive for lymphoma patients and negative for patients with lymphadenopathies due to other causes like tuberculosis or metastatic cancer. By the gel diffusion test with a sensitivity of 5–10 $\mu g\,ml^{-1}$ it has been possible to differentiate between lymphomas, other types of cancers and lymphadenitis.

*Development of radioimmunoassay*

The chloramine T method of Hunter was used for the trace iodination of LAA. About 60% of added $^{125}I$ was incorporated as revealed by the peaks of sephadex G-25 gel filtration (Figure 3). There were two peaks, a protein peak and an iodine peak. 1/1000 dilution of the antibody gave 50% binding. A linear relationship was observed between various concentrations of LAA (3.12–100 $ng\,ml^{-1}$) and the

**Figure 3** Purification of labelled LAA by Sephadex G-25 gel filtration.

**Figure 4** Standard curve of enzyme-labelled immunosorbent assay by indirect inhibition principle.

inhibition. The sensitivity of this assay was $15\,ng\,ml^{-1}$.

The sera of 17 normals and of 22 patients with other types of cancers did not inhibit at a dilution of 1:5 or 1:10 in this assay. The serum LAA levels for 24 lymphoma patients were in the sensitivity range of $187–1500\,ng\,ml^{-1}$. Similar results were obtained by the ELISA assay (Table IV). The standard inhibition curve was linear (Figure 4). Serial determinations of serum LAA levels for confirmed lymphoma patients showed a positive correlation to the course of the disease (Table V).

### Discussion

Using the xenogeneic antiserum raised in rabbits, the presence of a circulating LAA in lymphoma patients of T and B cell origin and of different histological types has been demonstrated. LAA has also been identified in the body fluids of lymphoma patients. The LAA test is virtually negative for normals and patients with various other types of cancers. The few false positive results obtained for non-lymphoid malignancies might be due to the presence of closely-related antigenic determinants present in the subpopulation of cells at different stages of differentiation. Recent studies on cell surface markers have shown a similarity between T cell acute lymphoblastic leukaemias and T cell lymphoblastic lymphomas (Nisson & Ponten, 1975; Minowada *et al.*, 1972). Immunological, cytological and physicochemical features suggest that these two lesions constitute a single disease process (Nathwani *et al.*, 1976; Pangalis *et al.*, 1979). An LAA test which is reactive for lymphoblastic lymphomas may also be reactive for lymphoblastic leukaemias.

Neoplastic cells derived from a single clone and residing in different anatomical compartments have been found to possess identical surface markers (Whiteside & Rowlands, 1977). Cell surface marker analysis has been found to be of particular use in determining whether the cytologically similar and dissimilar lymphoid proliferation occurring in different body sites are derived from the same malignant clone or otherwise (Minowada *et al.*, 1980). The serum LAA of different histological lymphomas may result from the same malignant clone.

Neoplastic B cell proliferations are reported to be heterogeneous with respect to SIg expression. Thus $Ia^+SIg^-$ and $Ia^+SIg^+$ cells may belong to the same neoplastic clone and represent cells at different stages of differentiation. This phenotypic heterogeneity has been demonstrated in T cell-derived malignant lymphoma also (Okamura *et al.*, 1981; Poppemma *et al.*, 1981). From these studies it is conceivable that a common antigen may exist for the various phenotypes of lymphomas or it may be that more than one binding site is present in the antibody molecule, each specific for T cell and B cell derived antigens or their subpopulations. This warrants further study.

A negative reaction with tissue extracts of other types of malignancies might indicate that LAA is perhaps a determinant selectively expressed by malignant lymphocytes.

The diagnostic potential of the LAA test appears promising since in a few patients with generalised lymphadenopathy, the test correlated well with histological diagnosis. Also for patients with no palpable lymph nodes the test had a lead time over the histological confirmation of a few months.

**Table IV** Determination of serum LAA levels by RIA and ELISA for controls and cancer patients*

| Normals and types of cancers | No. of cases | Serum LAA level ng $ml^{-1}$ RIA | ELISA |
|---|---|---|---|
| Controls | 17 | Undetectable | Undetectable |
| Hodgkin's Disease | | | |
| Nodular Sclerosis | 2 | 298,372 | 280,350 |
| Lymphocyte depletion | 4 | 568,734,1008,1500 | 524,698,930,1508 |
| Lymphocyte predominance | 3 | 328,294,505 | 272,255,496 |
| Mixed cellularity | 3 | 424,187,468 | 370,242,420 |
| Non Hodgkin's lymphoma | | | |
| Lymphocytic poorly differentiated | 3 | 770,483,442 | 715,440,395 |
| Lymphocytic intermediate differentiation | 3 | 328,296,334 | 288,254,272 |
| Lymphocytic well differentiated | 2 | 548,406 | 474,348 |
| Large lymphoid cells | 4 | 315,362,348,360 | 260,300,284,326 |
| Other types of cancer | 22 | Undetectable | Undetectable |

*The estimations were done in duplicate. The values are mean of 4 estimations of the same sample.

**Table V** Serial determinations of serum LAA levels for lymphoma patients.* (Preliminary data)

| Patient no. | Stage | Histological classification | Serum LAA ng $ml^{-1}$ At diagnosis | During treatment | At the end of therapy |
|---|---|---|---|---|---|
| 1. | I | Lymphocytic well differentiated | 410 | 326 | 78 |
| 2. | IV | Large lymphoid cells | 830 | 570 | 115 |
| 3. | II | Lymphocytic poorly differentiated | 152 | 96 | 48 |
| 4. | IV | Lymphocyte predominance** | 948 | 438 | 124 |
| 5. | II | Lymphocyte depletion** | 288 | 115 | 64 |
| 6. | II | Lymphocytic well differentiated | 376 | 220 | 45 |
| 7. | IIIB | Lymphocytic poorly differentiated | 540 | 265 | 82 |
| 8. | IV | Lymphocytic intermediate differentiation | 664 | 476 | 180 |
| 9. | II | Large lymphoid cells | 760 | 378 | 148 |

*These patients were treated by combination chemotherapy.
**Hodgkin's disease.

The presence of an LAA-anti LAA system in lymphomas has formed the basis for the development of more sensitive and specific assay procedures like RIA and ELISA for the early detection of these cancers.

The lower limit of detection by RIA is $15\,\text{ng}\,\text{ml}^{-1}$ and the upper limit $10\,\mu\text{g}\,\text{ml}^{-1}$ and has a sensitivity 100–1000 times greater than the double diffusion method. At this sensitivity range, LAA could not be detected in the sera of normals and of patients with other cancers. Hence this cut off may render this test relatively selective for lymphomas. The determination of serum LAA levels may serve as useful clinical index to follow the course of the disease. The serum LAA levels have been found to fluctuate during chemotherapy: significantly elevated levels observed initially fall gradually to very low levels at the end of effective treatment as seen from Table V.

Preliminary studies on the identification, quantitation and clinical evaluation of LAA have suggested the expression of a tumour-related antigen by malignant lymphocytes and the potential of this test for the detection of early malignant lymphomas. These results are being confirmed by preparing monoclonal antibodies to each histological type of lymphoma by the hybridoma technique of Köhler & Milstein as modified by Kennett (1981).

The authors gratefully acknowledge the grants received from the Council of Scientific and Industrial Research and the Department of Science and Technology, New Delhi.

**References**

AKIRA, Y., MATUSUUNA, Y., CARPENTER, C.M. & HYDE, L. (1968). Immunochemical studies on human lung cancer antigens soluble in 50% saturated ammonium sulphate. *J. Natl Cancer Inst.*, **40**, 663.

DAVIS, B.J. (1964). Disc electrophoresis II method and application to human serum proteins. *Ann. N.Y. Acad. Sci.*, **121**, 404.

GOLD, P. & FREEDMAN, S.O. (1965). Specific carcinoembryonic antigens of human digestive system. *J. Exp. Med.*, **122**, 467.

GREAVES, M.F. (1979). Immunodiagnosis of leukaemia In *Immunodiagnosis of Cancer* Part I. p. 542 (Eds. Herberman *et al.*) New York: Marcel Dekker, Inc.

HUNTER, W.M. (1967). The preparation of radioiodinated proteins of high activity, their reaction with antibody *in vitro*: the radioimmunoassay. In: *Handbook of Experimental Immunology,* (Ed. Weir) Blackwell Sci. Publ. Oxford, 608, 654.

KENNETT, R.H. (1981). Fusion protocols in Monoclonal antibodies: Hybridomas: A new dimension in biological analyses. p. 365, (Eds. Kennett & McKearn) Plenum press. New York.

LEABACK, D.H. (1975). *Isoelectric Focusing* p. 201, (Eds. Arbuthnott & Beeley) Butterworths. U.K.

MATHE, G., RAPPAPORT, H., O'CONOR, G.T. & TORLONI, H. (1976). *Histological and Cytological Typing of Neoplastic Diseases of Haematopoietic and Lymphoid Tissues.* World Health Organisation, Geneva.

MARREN, G.E. (1978). Enzyme immunoassays. Tijdschrift van de belgische vereniging van laboratoriumtechnologen. *Revue de l'Ass. Belge Technol. Lab.*, **5**, 199.

MINOWADA, J., SRIVASTAVA, B.I., FREEMAN, A.I. & SANDBERG, A.A. (1980). Heterogeneity of Marker profiles of 50 human leukemia lymphoma cell lines: A model for hematopoietic cell differentiation. *Proc. Am. Assoc. Cancer Res.*, **21**, 224.

MINOWADA, J., OHNUMA, T. & MOORE, G.E. (1972). Rosette-forming human lymphoid cell lines. I Establishment and evidence for origin of thymus-derived lymphocytes. *J. Natl Cancer Inst.*, **49**, 891.

MORTON, D.L., MALMGREN, R.A., HOLMES, E.C. & KETCHAM, A.S. (1968). Demonstration of antibodies against human malignant melanoma by immunofluorescence. *Surgery,* **64**, 233.

NATHWANI, B.N., KIM, H. & RAPPAPORT, H. (1976). Malignant lymphoma, lymphoblastic. *Cancer,* **38**, 964.

NISSON, K. & PONTEN, J. (1975). Classification and biological nature of established human haematopoietic cell lines. *Int. J. Cancer,* **15**, 321.

OKAMURA, S., CHECHIK, B.E., LEE, C., GELFAND, E.W. & MAK, T.W. (1981). Heterogeneity of human thymocytes and a malignant T-lympho-blast cell line. *Cancer Res.*, **41**, 1664.

ORDER, S.E., CHIM, S.E. & HELLMAN, S. (1973). Hodgkin's Disease associated antigen: Studies on segregation and specificities. *Natl Cancer Inst. Monogr.*, **36**, 139.

OUCHTERLONY, O. (1949). Antigen antibody reaction in gels. *Acta. Pathol. Microbiol. Scand.*, **26**, 507.

PANGALIS, G.A., NATHWANI, B.N., RAPPAPORT, H. & ROSEN, A.B. (1979). Acute lymphoblastic leukaemia. The significance of nuclear convolutions. *Cancer,* **43**, 551.

POPPEMMA, S., ELEMA, J.D. & HALIE, A.R. (1981). Alkaline phosphatase positive lymphomas: A morphologic, immunologic and enzyme-histochemical study. *Cancer,* **47**, 1303.

PORATH, J., AXEN, R. & ERNBACK, S. (1967). Chemical coupling of proteins to agarose. *Nature,* **215**, 1491.

TATARINOV, Y.S. (1964). Detection of embryo specific α-globulin in the blood sera of patients with primary liver tumour. *Vopr. Med. Khim.*, **10**, 90.

WHITESIDE, T.L. & ROWLANDS, T.D. (1977). T-cell and B-cell identification in the diagnosis of lympho-proliferative disease. A review. *Am. J. Pathol.*, **88**, 754.

YALLOW, R.S. & BERSON, S.A. (1960). Immunoassay of endogeneous plasma insulin in man. *J. Clin. Invest.*, **39**, 1157.